# To: Changes in respiratory mechanics during respiratory physiotherapy in mechanically ventilated patients

**DOI:** 10.5935/0103-507X.20150072

**Published:** 2015

**Authors:** Ângelo Roncalli Miranda Rocha, Caio Henrique Veloso da Costa

**Affiliations:** General Intensive Care Unit, Hospital Geral do Estado Professor Osvaldo Brandão Vilela - Maceió (AL), Brazil; Hospital Escola Hélvio Auto - Maceió (AL), Brazil; Centro de Estudos Superiores de Maceió - Maceió (AL), Brazil.; General Intensive Care Unit, Hospital Geral do Estado Professor Osvaldo Brandão Vilela - Maceió (AL), Brazil.

**To the Editor,**

We were very interested in the study by Moreira et al.^(1)^ as it reflects common and routine respiratory physiotherapy practices in intensive care units in Brazil and other countries. We appreciate the author's effort in examining the evidence for this type of therapy. In this study, an improvement was observed in the ventilatory mechanics parameters after the application of a respiratory physiotherapy protocol in patients dependent on mechanical ventilation. The authors report a significant increase in dynamic pulmonary compliance, tidal volume, and oxygen saturation and a reduction in respiratory system resistance after application of the protocol. This protocol consisted of chest compression and vibration maneuvers, 0.9% saline instillation, and hyperinflation with a manual resuscitator, followed by endotracheal aspiration. However, we note the absence of a control group to help determine whether these gains were due to the use of the protocol and whether these gains could not be achieved with the endotracheal suction procedure alone.

According to the AARC Clinical Practice Guidelines - Endotracheal Suctioning of Mechanically Ventilated Patients with Artificial Airways,^(2)^ the decrease in peak pressure and airway resistance and the increase in dynamic compliance and tidal volume are expected and desired outcomes for endotracheal suctioning procedures and, therefore, a confounding factor for the effectiveness of the proposed therapy.

The authors also did not report any peak airway pressure controls during hyperinflation with the manual resuscitator, which may compromise the safety of the procedure. Peak pressures above 40cmH_2_O may be associated with alveolar overdistention and the risk of barotrauma, suggesting, for safety reasons, the use of manometers coupled to a manual resuscitator during these maneuvers.^(3-5)^

*Ângelo Roncalli Miranda Rocha* *General Intensive Care Unit, Hospital Geral do Estado Professor Osvaldo
Brandão Vilela - Maceió (AL), Brazil; Hospital Escola Hélvio
Auto - Maceió (AL), Brazil; Centro de Estudos Superiores de
Maceió - Maceió (AL), Brazil.**Caio Henrique Veloso da Costa* *General Intensive Care Unit, Hospital Geral do Estado Professor Osvaldo
Brandão Vilela - Maceió (AL), Brazil.*
