# Supplementary material for: To: Changes in respiratory mechanics during respiratory physiotherapy in mechanically ventilated patients
Source: Rev Bras Ter Intensiva. 2015 Oct-Dec;27(4):422–3. doi: 10.5935/0103-507X.20150072 (PMC4738833; doi:10.5935/0103-507X.20150072)
Supplement: Related texts

# Reply: AUTHORS' RESPONSE

**Authors:** Cassiano Teixeira, Augusto Savi

**Affiliations:** Department of Clinical Medicine, Universidade Federal de Ciências da Saúde de Porto Alegre - Porto Alegre (RS), Brazil; Intensive Care Unit, Hospital Moinhos de Vento - Porto Alegre (RS), Brazil.; Intensive Care Unit, Hospital Moinhos de Vento - Porto Alegre (RS), Brazil.

We would like to thank you for your comments. Several respiratory physiotherapy techniques are used in critically ill patients.^(1)^ However, the evidence for the use of multimodal respiratory physiotherapy is conflicting.^(2)^ Even the Clinical Practice Guideline of an important respiratory care society is based on the experience of specialists and not on the results of randomized clinical trials because of lack of evidence.^(3)^

Regarding the first question raised, our study protocol followed a sequence of techniques. After mobilizing the secretions from the distal airways using compression and vibration maneuvers and manual hyperinflation, the method to remove the secretions focuses on tracheal aspiration. This, in turn, only succeeds in removing the secretions located up to the third bronchial generation. Thus, it should be preceded by the techniques used in this protocol. It would be inconsistent to not aspirate secretions removed from the distal airways to the proximity of the endotracheal tube because this could lead to patient-ventilator asynchrony, respiratory distress, endotracheal tube obstruction, and increased respiratory effort.

As noted in the letter, the simple act of performing tracheal aspiration can improve respiratory mechanics. However, this procedure should not be performed systematically because it can lead to complications including a decrease in dynamic pulmonary compliance and functional residual capacity, atelectasis, hypoxemia, a bronchoconstrictor response, and arrhythmias.^(4)^ The practical guide itself reports that this procedure, which is frequently used in patients on mechanical ventilation, can cause such complications.^(3)^ Therefore, we believe that the improvements in mechanical ventilation occurred because of the sequence of the procedures, which culminated in tracheal aspiration.

In response to the second question, we did not use a manometer for monitoring during the hyperinflation maneuver with a manual resuscitator because our equipment has a safety valve that prevents the airway pressure from exceeding 40cmH_2_O; therefore, there is no need to monitor the pressure using a manometer.

*Cassiano Teixeira* *Department of Clinical Medicine, Universidade Federal de Ciências
da Saúde de Porto Alegre - Porto Alegre (RS), Brazil; Intensive Care
Unit, Hospital Moinhos de Vento - Porto Alegre (RS), Brazil.**Augusto Savi* *Intensive Care Unit, Hospital Moinhos de Vento - Porto Alegre (RS),
Brazil.*

## References

[1] Gosselink R, Bott J, Johnson M, Dean E, Nava S, Norrenberg M (2008). Physiotherapy for adult patients with critical illness:
recommendations of the European Respiratory Society and European
Society of Intensive Care Medicine Task Force on Physiotherapy
for Critically Ill Patients. Intensive Care Med.

[2] Stiller K (2013). Physiotherapy in intensive care: an update systematic
review. Chest.

[3] American Association for Respiratory Care (2010). AARC Clinical Practice Guidelines. Endotracheal
suctioning of mechanically ventilated patients with artificial
airways 2010. Respir Care.

[4] Guglielminotti J, Alzieu M, Maury E, Guidet B, Offenstadt G (2000). Bedside detection of retained tracheobronquial
secretions in patients receiving mechanical ventilation: is it
time for tracheal suctioning?. Chest.
